# Fuzzy Clustering-Based Deep Learning for Short-Term Load Forecasting in Power Grid Systems Using Time-Varying and Time-Invariant Features

**DOI:** 10.3390/s24051391

**Published:** 2024-02-21

**Authors:** Kit Yan Chan, Ka Fai Cedric Yiu, Dowon Kim, Ahmed Abu-Siada

**Affiliations:** 1 School of Electrical Engineering, Computing and Mathematics Sciences, Curtin University, Bentley, WA 6102, Australia; dowon.kim@curtin.edu.au (D.K.); a.abusiada@curtin.edu.au (A.A.-S.); 2 Department of Applied Mathematics, The Hong Kong Polytechnic University, Hong Kong; macyiu@polyu.edu.hk

**Keywords:** deep neural network, smart sensors, fuzzy clustering, electric power forecasting, new customer demand forecasting

## Abstract

Accurate short-term load forecasting (STLF) is essential for power grid systems to ensure reliability, security and cost efficiency. Thanks to advanced smart sensor technologies, time-series data related to power load can be captured for STLF. Recent research shows that deep neural networks (DNNs) are capable of achieving accurate STLP since they are effective in predicting nonlinear and complicated time-series data. To perform STLP, existing DNNs use time-varying dynamics of either past load consumption or past power correlated features such as weather, meteorology or date. However, the existing DNN approaches do not use the time-invariant features of users, such as building spaces, ages, isolation material, number of building floors or building purposes, to enhance STLF. In fact, those time-invariant features are correlated to user load consumption. Integrating time-invariant features enhances STLF. In this paper, a fuzzy clustering-based DNN is proposed by using both time-varying and time-invariant features to perform STLF. The fuzzy clustering first groups users with similar time-invariant behaviours. DNN models are then developed using past time-varying features. Since the time-invariant features have already been learned by the fuzzy clustering, the DNN model does not need to learn the time-invariant features; therefore, a simpler DNN model can be generated. In addition, the DNN model only learns the time-varying features of users in the same cluster; a more effective learning can be performed by the DNN and more accurate predictions can be achieved. The performance of the proposed fuzzy clustering-based DNN is evaluated by performing STLF, where both time-varying features and time-invariant features are included. Experimental results show that the proposed fuzzy clustering-based DNN outperforms the commonly used long short-term memory networks and convolution neural networks.

## 1. Introduction

Power grid systems supply power loads for millions of users which are dynamic and complex. Therefore, efficient and reliable power grid systems are essential for maintaining power stability and avoiding power system outages and supply user load demands without power interruptions [1,2]. A sufficient power utilization scheme with accurate short-term load forecasting (STLF) is necessary for application on power grid systems [3,4,5]. One percent of forecasting error can cause operation losses of 10 million or more [6]. Since 40% of electrical power is supplied to buildings through the power grid system, an accurate STLF benefits all stakeholders of the energy market and results in substantial savings for users [7]. An accurate STLP also contributes significant savings economically and also ensures power grid reliability and security [8]. Accurate forecasting is essential for the system controller to maintain grid system stability [9,10,11,12,13].

To perform STLF, physics-based models consisting of system equations can be used. Those physics-based models can be used to explicitly illustrate the system dynamics. However, developing those physics-based models requires extensive knowledge of the internal components of systems or buildings which are related to power consumption. Data-driven models are developed by data. Developing such models does not require extensive knowledge relating to systems or buildings. Thanks to advanced smart sensor technologies, smart meters can be used to capture the loads consumed by users in real time. Smart sensors can be used to capture weather information such as temperature, wind speed/direction and sea level pressure, which are correlated with load consumption. This time-series data is captured in real time in order to perform accurate and reliable STLF [14,15]. Among those data-driven models, deep neural networks (DNNs) have commonly been used, since DNNs consist of complex, multiple-neuron layers which are effective for modelling nonlinear and chaotic load consumption data [16]. Recent DNN techniques for STLP can be classified into two categories, (i) single time-varying feature, where the DNN uses past load consumption to predict future load consumption, and (ii) multi-time-varying features, where the DNN uses past dynamic information such as past weather conditions, past meteorology information, past seasonal and calendar information to predict future load consumption, despite using past load consumption.

For the single time-varying feature, the DNNs forecast future load consumption using past load consumption. A long short-term memory (LSTM)-based DNN was developed using past load consumption sequences of appliances in order to forecast future load consumption [17]. Peng et al. [18] applied linear regression and LSTM to forecast future load consumption using past load consumption. Hafeez et al. [19] proposed a Boltzmann machine-based DNN to predict future load consumption using past load consumption. Aly et al. [20] proposed a clustering technique which used past load consumption in order to classify the future load consumption demands of users. Based on past power demands, various models have been developed to predict load consumption for various users. Rafati et al. [21] proposed a dense neural network to model the nonlinear and dynamic characteristics of past electrical load in order to predict future load consumption. Sekhar et al. [22] proposed a hybrid DNN by combining LSTM and a convolution neural network (CNN) to perform load prediction using past load information. Hybrid DNNs based on CNN, LSTM and decision tree have been proposed by Wan et al. [23] and Massaoudi et al. [24] to improve prediction accuracy. Tavassoli-Hojati et al. [25] proposed a self-partitioning local neuro-fuzzy model, where the model is trained by analysing both the linear and nonlinear characteristics of past load time-series features. Wei et al. [26] proposed a decomposition algorithm based on detrend singular spectrum fluctuation analysis to extract the trend and periodic components in past load data. An LSTM was trained with the extracted components. Yang et al. [27] proposed a decomposition approach to extract the time-series components of past load consumption. The decomposition approach captures useful past load consumption components to train DNNs.

For the multi-time-varying features, DNN models are developed by correlating future load consumption with past load consumption and past dynamic information such as past seasonal time information, past weather or meteorological conditions. Liang et al. [28] developed a hybrid DNN based on empirical mode decomposition and a regression neural network; the features used in the DNN included past temperature, past meteorology conditions and past load consumption. Ahmad et al. [29] proposed a novel DNN which included the features of past load information and past meteorology conditions. Kwon et al. [30] proposed a DNN where both past weather information and past load consumption were used as the DNN inputs. An adaptive neuro fuzzy inference system was proposed to predict the future load consumption of the Rajasthan region of India using past load consumption and past acute climatic conditions [31]. Zor et al. [32] proposed a DNN where the DNN inputs were based on past load consumption and past meteorological variables at a large hospital in the eastern Mediterranean. Eseye et al. [33] developed a hybrid machine-learning technique where the features included past weather, past load consumption, past seasonality and calendar information. Eseye et al. [34] proposed a novel feature selection based on a genetic algorithm to select significant features to improve load consumption forecasting accuracy. Hu et al. [35] proposed a back propagation-based neural network to predict the load consumption of the process industry where past load consumption, past production planning information and past humidity were used as DNN inputs. Yaprakdal et al. [36] proposed a feedforward neural network to predict the future load consumption, where the time-varying features included past load consumption, past temperature, past direct horizontal radiation and past diffuse horizontal radiation. Tziolis et al. [37] proposed a Bayesian neural network model where time-varying features such as past load consumption, past humidity, past dew point temperature, past horizontal irradiance and past wind speed were used as the network inputs.

The aforementioned DNN models only use the dynamics of single time-varying features or multi-time-varying features in order to forecast future load consumption. They use those dynamic features as the DNN inputs. They do not use the static information of the time-invariant features such as year built, building spaces, number of person in the building or building purposes. In fact, these time-invariant features are related to load consumption. When both time-invariant and time-varying features are used as the DNN inputs, more information is available for the DNNs to perform STLF; therefore, more accurate predictions are likely to be achieved. For example, building ages relate to load consumption [38]. Newer buildings consume less energy since they are constructed with strong isolation material. Older buildings consume more energy since the isolation material is generally poorer than in new buildings. More electricity for heaters or air-conditioners is consumed. As another example, more electricity is used for a larger building space, while less electricity is consumed for a smaller building. Hence, building space correlates to load consumption [39]. Buildings with more users consume more energy; less energy is consumed for buildings with a smaller number of users [40,41]. Occupant characteristics such as age, education, income and residency length are also correlated to load consumption [42,43]. On the contrary, building purposes relate to power consumption. Commercial or industrial buildings use more energy; resident buildings use less energy [44]. When more correlated features are included, more accurate predictions are likely to be achieved. Therefore, we can use time-invariant features to improve STLF since time-invariant features are also correlated to load consumption.

In this paper, a fuzzy clustering-based DNN is proposed by using both time-varying and time-invariant features to perform STLF. Clusters are generated to classify users with respect to time-invariant features, where the fuzzy c-means algorithm [45] is used since this algorithm is commonly used to cluster samples with time-invariant features [46]. Each cluster groups old users with similar time-invariant features which address the static information. Various DNNs are developed by the time-varying features of old users in the corresponding clusters, which have similar time-invariant features. The time-varying features of users in the same cluster are shared and are used to develop a DNN model for this particular cluster. Since the time-invariant features are already used to cluster users, the DNN model does not need to include the time-invariant features and the model is simpler. In addition, the DNN model only needs to learn time-varying features and it predicts time-varying dynamics for users in the same cluster, which has similar time-invariant features. Therefore, more accurate predictions of time-varying dynamics are likely to be achieved by the proposed model, compared to the commonly used DNN models, which need to address time-varying dynamics for all users. The proposed fuzzy clustering-based DNN is integrated with an LSTM and a CNN which is commonly used for STLF when time-varying features are used [17,28,29,30,31,33,34,36]. The performance of the proposed fuzzy clustering-based DNN was evaluated by Miller’s data [47,48], which includes both time-varying features such as load consumption, air temperature and wind speed and invariant time features such as building size and floor count. Experimental results show that more accurate forecasting can be achieved by the proposed fuzzy clustering-based DNN to predict the load consumption of new users when the data of new users is not available to train the DNN.

The main contributions of this research article are listed below.

(1) To perform STLF, the existing approaches only use time-varying dynamics such as past load consumption or past power correlated features [46,49,50,51,52,53,54]. No existing approach uses time-invariant features such as building spaces or building age to perform STLF. A novel approach is proposed in this paper to incorporate both time-varying and time-invariant features in order to improve STLF accuracy.
(2) A novel STLF approach, namely fuzzy clustering-based DNN, is proposed by incorporating fuzzy clustering and deep learning. The fuzzy clustering addresses time-invariant features and the deep learning addresses time-varying features. This incorporation improves existing DNN models, which only address time-varying features.
(3) The proposed fuzzy clustering-based DNN is evaluated by Miller’s dataset [47,48], which is used for evaluating load consumption predictors. The datasets are involved with both time-invariant and time-varying features. The results demonstrate that better STLF can be achieved by the proposed fuzzy clustering-based DNN.
(4) To evaluate the prediction performance of the proposed fuzzy clustering-based DNN, its prediction performance is compared with some recently published STLF approaches.

The rest of the article is structured as follows: Section 2 describes the purposes of STLF and describes how a DNN model can be developed for STLF. Section 3 describes the mechanism of the proposed fuzzy clustering-based DNN. It also describes how the fuzzy clustering addresses the time-invariant features and the DNN model addresses the time-varying features. Section 4 shows the load consumption data, which is used for evaluating the proposed method; it shows how the proposed method is implemented, and the prediction results are also shown, compared with other existing methods. A conclusion is drawn in Section 5.

## 2. Load Consumption Forecasting

The STLF performed by the DNN model is given as (1):

(1) $$\hat{x}\left(t+m\right)=\Theta \left(\bar{F}\left(t-p,t\right),W\right)+e\left(t+m\right)$$

where

(2) $$\begin{array}{cc} \; & \bar{F}\left(t-p,t\right)=\left\{\left[x\left(t\right),\bar{y}\left(t\right)\right],\left[x\left(t-1\right),\bar{y}\left(t-1\right)\right],\ldots ,\left[x\left(t-p\right),\bar{y}\left(t-p\right)\right]\right\}\\ \; & \bar{y}\left(t-k\right)=\left[y_{1}\left(t-k\right),y_{2}\left(t-k\right),\ldots ,y_{N}\left(t-k\right)\right]\;\mathrm{with}\;k=0,1,\ldots ,p \end{array}$$

In (1), the DNN model, Θ, forecasts future load consumption, $\hat{x}\left(t+m\right)$, with *m* time samples ahead. *W* is the parameter set of Θ, which needs to be optimized with respect to the prediction accuracy. e(t+m) is the noise residual at time (t+m). $\bar{F}\left(t-p,t\right)$ in (2) is the past information set, which is windowed by a time series between the current time, *t*, to the past, *p*, samples of time. $\bar{y}\left(t-k\right)$ with k=0,1,…,p denotes the forecasting feature vector which contains the $i^{th}$ forecasting feature, $y_{i}\left(t-k\right)$ with i=1,2,…,N, such as past weather information, past climate information, past seasonal information, user information and building information. x(t−k) is the past load consumption. Both x(t−k) and $\bar{y}\left(t-k\right)$ are correlated to the future load consumption. Therefore, $\bar{F}\left(t-p,t\right)$, containing both x(t−k) and $\bar{y}\left(t-k\right)$, is used to forecast $\hat{x}\left(t+m\right)$.

To optimize Θ, *W* is determined by the training dataset collected from the *M* existing users, namely $D=[d^{\left(1\right)},d^{\left(2\right)},\ldots ,d^{\left(M\right)}]$, where $d^{\left(i\right)}$ in (3) is the data collected for the $i^{th}$ user with i=1,2,…,M, which contains *n* samples of past load consumption and the past information set.

(3) $$d^{\left(i\right)}=\left\{\left[{\bar{F}}^{\left(i\right)}\left(t-p-j,t-j\right),x^{\left(i\right)}\left(t+m-j\right)\right]\;\mathrm{with}\;j=0,1,\ldots ,\left(n-1\right)\right\}$$

where ${\bar{F}}^{\left(i\right)}\left(t-p-j,t-j\right)$ is the past information set windowed with time (t−p−j) to (t−j) for the $i^{th}$ user; $x^{\left(i\right)}\left(t+m-j\right)$ is the load consumption at time (t+m−j) for the $i^{th}$ user. ${\bar{F}}^{\left(i\right)}\left(t-p-j,t-j\right)$ is further written as:

(4) $$\begin{array}{cc} \; & {\bar{F}}^{\left(i\right)}\left(t-p-j,t-j\right)=\{\left[x^{\left(i\right)}\left(t-k-j\right),\left[y_{1}^{\left(i\right)}\left(t-k-j\right),y_{2}^{\left(i\right)}\left(t-k-j\right),\ldots ,y_{N}^{\left(i\right)}\left(t-k-j\right)\right]\right]\;\\ \; & \mathrm{with}\;k=0,1,\ldots ,p\} \end{array}$$

which contains the past load consumption and past forecasting features within the time window between (t−p−j) and (t−j) for the $i^{th}$ user.

Based on the past information set and the load consumption in *D*, *W* in Θ can be determined by solving the optimization problem in (5).

(5) $$\min_{W}\sum_{i=1}^{M}\sum_{j=0}^{\left(n-1\right)}{\left(\Theta \left({\bar{F}}^{\left(i\right)}\left(t-p-j,t\right),W\right)-x^{\left(i\right)}\left(t+m-j\right)\right)}^{2}$$

**Figure 1:**
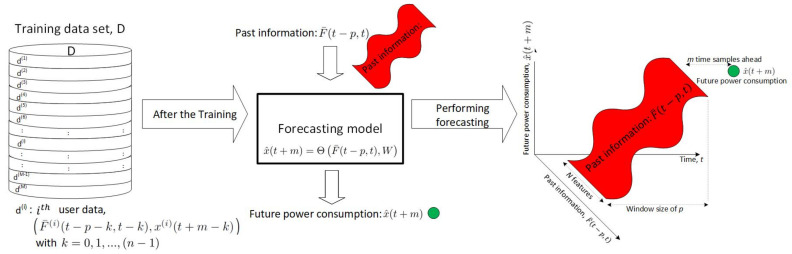
Forecasting framework.

The forecasting framework is shown in Figure 1. The DNN model, Θ, is developed by the training dataset, *D*, which contains the data from the *M* users, $d^{\left(1\right)},d^{\left(2\right)},\ldots ,d^{\left(M\right)}$. Some past information features are time-invariant, such as building spaces, year built, number of building floors and building purposes. Those time-invariant features are related to load consumption for new users. We can use those time-invariant features to improve the prediction accuracy for new users. For example, a larger building space uses more electricity, while less electricity is consumed with a smaller building. In addition, building age correlates with energy consumption, since older buildings are mostly constructed with older material which has less isolation capability. Hence, more energy is required to warm or cool buildings during winters or summers. For modern buildings, better isolation material is used and less energy is consumed. Furthermore, building purposes are related to user behaviours regarding power consumption. Residential buildings use more energy at night time and less energy at day time. On the contrary, commercial or industrial buildings use more energy at day time and less energy at night time. Therefore, building space, building age and building purpose are time-invariant features which correlate to load consumption. Section 3 discusses how time-invariant features are used to improve STLF.

## 3. Fuzzy Clustering-Based Deep Learning Model

All forecasting features, $y_{i}\left(t\right)\in \bar{y}\left(t\right)$ in (2), with i=1,2,…,N are divided by two sets of features in (6), namely time-invariant features, ${\bar{y}}_{I}$, and time-varying features, ${\bar{y}}^{v}\left(t\right)$, where *C* is the number of time-invariant features and (N−C) is the number of time-varying features. All elements in ${\bar{y}}_{I}$ are constants since they are time-invariant.

(6) $$\begin{array}{cc} \; & \;\;\bar{y}\left(t\right)=\left[{\bar{y}}_{I},{\bar{y}}_{V}\left(t\right)\right]\\ \; & \mathrm{with}\;{\bar{y}}_{I}=\left(y_{1},y_{2},\ldots ,y_{C}\right)\\ \; & \;\;{\bar{y}}_{V}\left(t\right)=\left(y_{C+1}\left(t\right),y_{C+2}\left(t\right),\ldots ,y_{N}\left(t\right)\right) \end{array}$$

Given that the first *C* features are time-invariant constants, the past information of the $i^{th}$ user in (4) can be rewritten as:

(7) $$\begin{array}{cc} \; & {\bar{F}}^{\left(i\right)}\left(t-p,t\right)=\{\left[x^{\left(i\right)}\left(t-k\right),\left[y_{1}^{\left(i\right)},y_{2}^{\left(i\right)},\ldots ,y_{C}^{\left(i\right)},y_{\left(C+1\right)}^{\left(i\right)}\left(t-k\right),y_{\left(C+2\right)}^{\left(i\right)}\left(t-k\right),\ldots ,y_{N}^{\left(i\right)}\left(t-k\right)\right]\right]\;\\ \; & \mathrm{with}\;k=0,1,\ldots ,p\} \end{array}$$

Substituting ${\bar{y}}_{I}^{\left(i\right)}$ and ${\bar{y}}_{V}^{\left(i\right)}\left(t-k\right)$ into (7), ${\bar{F}}^{\left(i\right)}\left(t-p,t\right)$ can be rewritten as:

(8) $${\bar{F}}^{\left(i\right)}\left(t-p,t\right)=\left\{\left[x^{\left(i\right)}\left(t-k\right),\left[{\bar{y}}_{I}^{\left(i\right)},{\bar{y}}_{V}^{\left(i\right)}\left(t-k\right)\right]\right]\;\mathrm{with}\;k=0,1,\ldots ,p\right\}$$

where the terms with the subscripts from 1 to *C* in (7) are the time-invariant feature data for the $i^{th}$ user. The terms are included in a vector, ${\bar{y}}_{I}^{\left(i\right)}=\left(y_{1}^{\left(i\right)},y_{2}^{\left(i\right)},\ldots ,y_{C}^{\left(i\right)}\right)$; those from (C+1) to *N* are the time-varying feature data, which is written as a vector, ${\bar{y}}_{V}^{\left(i\right)}\left(t-k\right)=\left(y_{C+1}^{\left(i\right)}\left(t-k\right),y_{C+2}^{\left(i\right)}\left(t-k\right),\ldots ,y_{N}^{\left(i\right)}\left(t-k\right)\right)$ with k=0,1,…,p.

The time-varying set for the $i^{th}$ user is grouped as:

(9) $${\bar{Y}}_{V}^{\left(i\right)}=\left[{\bar{y}}_{V}^{\left(i\right)}\left(t\right),{\bar{y}}_{V}^{\left(i\right)}\left(t-1\right),\ldots ,{\bar{y}}_{V}^{\left(i\right)}\left(t-p\right)\right]$$

In this section, a fuzzy clustering-based DNN model is proposed to forecast the load consumption of new users. Clusters are generated to classify users with respect to time-invariant features using the time-invariant vector ${\bar{y}}_{I}^{\left(i\right)}$ with i=1,2,…,M. Each cluster is grouped with users with similar time-invariant features. Each DNN model is developed by time-varying sets for users in the same cluster, which have similar time-invariant features. Hence, all ${\bar{Y}}_{V}^{\left(i\right)}$ in the same cluster are used to develop a DNN model. The time-varying features in the same cluster are shared and are used to develop the DNN model.

Since the time-invariant features are already used to cluster users, the DNN model does not need to include the time-invariant features and the model only uses the time-varying features to forecast future load consumption; therefore, a simpler model can be generated. In addition, the model only needs to learn the time-varying features and predict time-varying dynamics in the clusters which have similar time-invariant features. Therefore, the learning is simpler and more accurate predictions of time-varying dynamics are likely to be achieved by the proposed model, compared to the commonly used DNN models which address both the time-varying and time-invariant dynamics. Section 3.1 discusses the clustering method for classifying users based on time-invariant features. Section 3.2 discusses the deep-learning models based on time-varying features to forecast future load consumption.

### 3.1. Clustering of Time-Invariant Features

When the time-invariant vectors of all users are given, clusters can be generated to classify users which have similar behaviours of using electrical power. Given that we have $N_{c}$ clusters with $2\leq N_{c}\leq M$, we determine which cluster the $i^{th}$ user belongs to, where i=1,2,…,M. Here, ${\hat{u}}_{k}\left({\bar{y}}_{I}^{\left(i\right)}\right)$ in (10) is defined as the membership of the $i^{th}$ user to the $k^{th}$ cluster, where $k=1,2,..,N_{C}$. The membership indicates how much ${\bar{y}}_{I}^{\left(i\right)}$ belongs to the $k^{th}$ cluster. If ${\hat{u}}_{k}\left({\bar{y}}_{I}^{\left(i\right)}\right)$ is large, the $i^{th}$ user has a similar behaviour to the users in the $k^{th}$ cluster. Therefore, ${\bar{y}}_{I}^{\left(i\right)}$ is in the $k^{th}$ cluster if ${\hat{u}}_{k}\left({\bar{y}}_{I}^{\left(i\right)}\right)>{\hat{u}}_{j}\left({\bar{y}}_{I}^{\left(i\right)}\right)$ for all $k\neq j\in 1,2,\ldots ,N_{c}$.

(10) $$\begin{array}{c} {\hat{u}}_{k}\left({\bar{y}}_{I}^{\left(i\right)}\right)={\left(\sum_{j=1}^{N_{c}}{\left(\frac{d_{ik}}{d_{ij}}\right)}^{\frac{2}{\left(m_{f}-1\right)}}\right)}^{-1} \end{array}$$

where $d_{ik}$ is the $\bar{A}$ norm distance between ${\bar{y}}_{I}^{\left(i\right)}$ and the $k^{th}$ cluster centre, and $m_{f}$ is the weighting exponent with $1\leq m_{f}<\infty$. $d_{ik}$ is given as:

(11) $$d_{ik}=\left|\right|{\bar{y}}_{I}^{\left(i\right)}-{\bar{v}}_{k}{\left|\right|}_{\bar{A}}^{2}={\left({\bar{y}}_{I}^{\left(i\right)}-{\bar{v}}_{k}\right)}^{T}\bar{A}\left({\bar{y}}_{I}^{\left(i\right)}-{\bar{v}}_{k}\right)$$

where $\bar{A}$ is a positive definite n×n weight matrix and ${\bar{v}}_{k}$ denotes the centre of the $k^{th}$ cluster, which is given by:

(12) $$\begin{array}{c} {\bar{v}}_{k}=\frac{\sum_{i=1}^{M}{\left({\hat{u}}_{k}\left({\bar{y}}_{I}^{i}\right)\right)}^{m_{f}}\times {\bar{y}}_{I}^{\left(i\right)}}{\sum_{i=1}^{M}{\left({\hat{u}}_{k}\left({\bar{y}}_{I}^{i}\right)\right)}^{m_{f}}};\;\mathrm{with}\;1\leq k\leq N_{c} \end{array}$$

To determine the cluster centres, $\bar{V}=\left({\bar{v}}_{1},{\bar{v}}_{2},\ldots ,{\bar{v}}_{N_{c}}\right)$, the generalized least-squared error in (13) is minimized for all ${\bar{y}}_{I}^{\left(i\right)}$ [45].

(13) $$J_{C-fuzz}\left(\bar{V}\right)=\sum_{k=1}^{N_{c}}\sum_{i=1}^{M}d_{ik}^{2}\times {\left({\hat{u}}_{k}\left({\bar{y}}_{I}^{\left(i\right)}\right)\right)}^{m_{f}}$$

In (13), ${\hat{u}}_{k}\left({\bar{y}}_{I}^{\left(i\right)}\right)$ is the membership function of ${\bar{y}}_{I}^{\left(i\right)}$ to the $k^{th}$ cluster and $d_{ik}$ is the $\bar{A}$-norm distance between the $i^{th}$ user to the $k^{th}$ cluster centre. The weight attached to each $d_{ik}$ is ${\left({\hat{u}}_{k}\left({\bar{y}}_{I}^{\left(i\right)}\right)\right)}^{m_{f}}$, which is the $m_{f}$ power of the ${\bar{y}}_{I}^{\left(i\right)}$ membership in cluster *k*. Therefore, minimizing (13) ensures that all users are close to their corresponding cluster centres. If $m_{f}=1$, $J_{C-fuzz}$ minimizes equally to all distances. If $m_{f}$ is larger, $J_{C-fuzz}$ minimizes large distances since the power of large distances dominates other small distances.

To minimize $J_{C-fuzz}\left(\bar{V}\right)$, the FCM algorithm is proposed [45]. The FCM algorithm is one of the most commonly used methods for identifying cluster centres and memberships between each sample to each cluster. Recent research shows that the FCM algorithm is an effective approach for clustering data [46,49], particularly in solving recent engineering problems such as predicting power system risks [50], bearing fault diagnosis [55], power equipment image segmentation [51], PV array fault diagnosis [52] and classifying load consumption for users [53], classifying groundwater quality [54]. Therefore, we proposed the FCM algorithm illustrated in Algorithm 1 to minimize (13) in order to determine the optimal cluster centres, $\bar{V}=\left\{{\bar{v}}_{1},{\bar{v}}_{2},\ldots ,{\bar{v}}_{N_{c}}\right\}$, in (12). The fuzzy partition coefficient, $V_{pc}$, indicates the clustering performance.

In the FCM algorithm, the inputs are the time-invariant features of the *M* users. The first two steps randomly initialize a membership matrix which indicates how much a user belongs to a cluster. Step 3 initializes the first set of cluster centres using (12). Step 5 computes the membership of a user to a cluster using (10), and it generates the membership matrix. Step 6 compares whether the membership matrix is smaller than a threshold. If the membership matrix is smaller, the fuzzy partition coefficient is computed; both the computed fuzzy partition coefficient and the computed cluster centres in Step 3 are returned as the output of the FCM algorithm. Otherwise, Step 3 computes the cluster centres and the algorithm is repeated iteratively.
**Algorithm 1** 
Fuzzy C-Means (FCM) Algorithm
**Input:** All time-invariant vectors ${\bar{y}}_{I}^{\left(i\right)}$, with i=1,2,…,M.**Output:** The centres of the clusters, $\bar{V}=\left\{{\bar{v}}_{1},{\bar{v}}_{2},\ldots ,{\bar{v}}_{N_{c}}\right\}$; the fuzzy partition coefficient, $V_{pc}$  **Step 1:** Set the algorithmic parameters, $N_{c}$, *m*, $\bar{A}$, and the threshold, ϵ.  **Step 2:** Randomly initialize a membership matrix, ${\hat{U}}^{iter}=\left[{\hat{u}}_{ik}^{iter}\right]$, with             the iteration iter=1.  **Step 3:** Compute the cluster centres $\bar{V}=\left\{{\bar{v}}_{1},{\bar{v}}_{2},\ldots ,{\bar{v}}_{N_{c}}\right\}$ using (12).  **Step 4:** Set iter=iter+1.  **Step 5:** Compute an updated membership matrix, ${\hat{U}}^{iter}=\left[{\hat{u}}_{ik}^{iter}\right]$,            with ${\hat{u}}_{ik}^{iter}={\hat{u}}_{k}\left({\bar{y}}_{I}^{\left(i\right)}\right)$ using (10).  **Step 6:** Compare whether ${\hat{U}}^{iter}$ is higher than ${\hat{U}}^{iter-1}$:                  **If** $\left|\right|{\hat{U}}^{iter}-{\hat{U}}^{iter-1}\left|\right|$ is smaller than ϵ,                        **then** compute the fuzzy partition coefficient, $V_{pc}$,                              $V_{pc}=\frac{1}{M}\sum_{i=1}^{N_{c}}\sum_{j=1}^{M}{\left(\hat{u_{ij}^{iter}}\right)}^{2}$                        **goto Step 7**.                  **Else** Set ${\hat{U}}^{iter-1}$ as ${\hat{U}}^{iter}$ and goto **Step 3**.  **Step 7: Return** The cluster centres, $\hat{V}$

After the cluster centres are determined, they are used to determine the memberships to each cluster when the time-invariant vector ${\bar{y}}_{I}^{\left(i\right)}$ of the $i^{th}$ user is given. The $i^{th}$ user belongs to the $k^{th}$ cluster if the membership belonging to the $k^{th}$ cluster is larger than that belonging to the $j^{th}$ cluster, where

(14) $${\hat{u}}_{k}\left({\bar{y}}_{I}^{\left(i\right)}\right)>{\hat{u}}_{j}\left({\bar{y}}_{I}^{\left(i\right)}\right)$$

with j≠k=1,2,…,M, and the membership of ${\bar{y}}_{I}^{\left(i\right)}$ to the $k^{th}$ cluster is

(15) $${\hat{u}}_{k}\left(y_{I}^{\left(i\right)}\right)=\sum_{i=1}^{N_{c}}\left(\frac{{\left({\bar{y}}_{I}^{\left(i\right)}-{\bar{v}}_{k}\right)}^{T}\bar{A}\left({\bar{y}}_{I}^{\left(i\right)}-{\bar{v}}_{k}\right)}{{\left({\bar{y}}_{I}^{\left(i\right)}-{\bar{v}}_{j}\right)}^{T}\bar{A}\left({\bar{y}}_{I}^{\left(i\right)}-{\bar{v}}_{j}\right)}\right)$$

Each ${\bar{y}}_{I}^{\left(i\right)}$ belongs to one of the *M* clusters. The time-varying sets of all users in a single cluster are used to develop a model to predict the future load consumption. ${\bar{Y}}_{V}^{\left(p(j,k)\right)}$ with $k=1,2,\ldots ,O_{j}$ are in the $j^{th}$ cluster, where ${\hat{p}}_{j}$ denotes the index vector which indicates the time-invariant vectors in the $j^{th}$ cluster.

(16) $${\hat{p}}_{j}=\left\{p\left(j,1\right),p\left(j,2\right),\ldots ,p\left(j,O_{j}\right)\right\}$$

where $O_{j}$ is the number of elements in the $j^{th}$ cluster and the $p{\left(j,k\right)}^{th}$ time-invariant vector with $k=1,2,\ldots ,O_{j}$ is in the $j^{th}$ cluster. All p(j,k) in ${\hat{p}}_{j}$ are different, where 1≤p(j,k)≤M. Since there are $N_{c}$ clusters, $N_{c}$ models are developed using the time-varying sets.

Fuzzy Deep Learning in Algorithm 2 illustrates how the $N_{c}$ models are developed, when the time-invariant vectors and time-varying sets are given. The first two steps generate $N_{c}$ cluster centres using the FCM in Algorithm 1. Step 3 determines the time-invariant vector belonging to each cluster, based on (15). Step 4 determines the index vector of time-varying sets to each cluster using (16). Step 5 develops the model using the time-varying sets in each cluster. Each model is developed based on the time-varying sets in the corresponding cluster. In this paper, the two commonly used deep-learning approaches, namely LSTM and CNN described in Section 3.2.1 and Section 3.2.2, are used, respectively.
**Algorithm 2** 
Fuzzy Deep learning
**Input:** All time-invariant vector ${\bar{y}}_{I}^{\left(i\right)}$ and time-varying set ${\bar{Y}}_{V}^{\left(i\right)}$, with i=1,2,…,M.**Output:** $N_{c}$ DNN models, $\Theta_{i}$, with $i=1,2,\ldots ,N_{c}$, which forecasts future                load consumption.  **Step 1:** Initialize the parameters, $N_{c}$, *m*, $\bar{A}$, and $\left|\right|\cdot {\left|\right|}_{\bar{A}}$, and the threshold, ϵ.  **Step 2:** Generate the $N_{c}$ cluster centres, $\bar{V}=\left\{{\bar{v}}_{1},{\bar{v}}_{2},\ldots ,{\bar{v}}_{N_{c}}\right\}$, using            the FCM in Algorithm 1.  **Step 3:** Determine the membership of ${\bar{y}}_{I}^{\left(i\right)}$ to the $k^{th}$ cluster, ${\hat{u}}_{k}\left(y_{I}^{\left(i\right)}\right)$ using (15).  **Step 4:** Determine the index vector, ${\hat{p}}_{j}=\left\{p\left(j,1\right),p\left(j,2\right),\ldots ,p\left(j,O_{j}\right)\right\}$ using (16),            with j=1,2,…,M, which indexed the time-varying sets in the $j^{th}$ cluster.  **Step 5:** Use all time-varying sets ${\bar{Y}}_{V}^{p(j,k)}$ with $k=1,2,\ldots ,O_{j}$ to develop the            DNN model, $\Theta_{j}$, using deep learning.  **Step 6: Return** The DNN models $\Theta_{j}$ with $j=1,2,\ldots ,N_{c}$.

**Figure 2:**
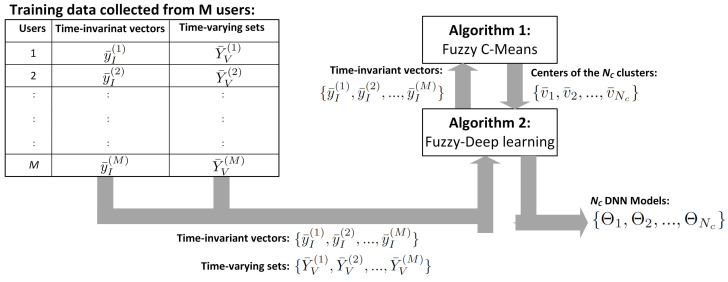
Training fuzzy clustering-based DNN.

The flow involving FCM in Algorithm 1 and Fuzzy Deep learning in Algorithm 2 is summarized in Figure 2. FCM generates the centres of the $N_{c}$ clusters using the time-invariant vectors; Fuzzy Deep Learning generates the $N_{c}$ DNN models using the time-varying sets. As aforementioned in Section 1, existing methods only use time-varying sets to develop DNN models for STLF. In fact, DNN models can be trained by both time-invariant vectors and time-varying sets, when both are available. The number of inputs in the DNN models is more than that of the proposed fuzzy clustering-based DNN, since the proposed fuzzy clustering-based DNN is only trained by the time-varying sets. Therefore, the proposed fuzzy clustering-based DNN is simpler than the existing DNN models.

**Figure 3:**
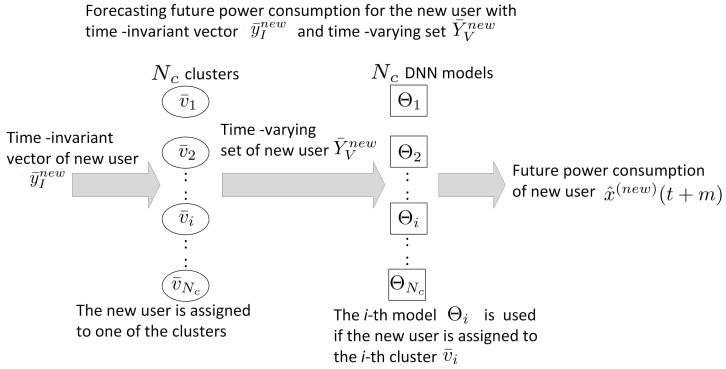
Implementing fuzzy clustering-based DNN after the training.

After the $N_{c}$ cluster centres and the $N_{c}$ models are generated, the fuzzy clustering-based DNN in Figure 3 can be used to forecast future load consumption when the time-invariant vector and time-varying set of a new user, namely ${\bar{y}}_{I}^{new}$ and ${\bar{Y}}_{V}^{new}$, are given. We assume that the membership of ${\bar{y}}_{I}^{new}$ belonging to the $i^{th}$ cluster is larger than that belonging to the other clusters. The new user belongs to the $i^{th}$ cluster with the cluster centre ${\bar{v}}_{i}$. The corresponding $i^{th}$ model, $\Theta_{i}$, uses ${\bar{Y}}_{V}^{new}$ to predict the future load consumption, ${\hat{x}}^{new}\left(t+m\right)$. If the membership is smaller than a threshold value, the DNN trained by both time-varying sets and time-invariant vectors is used. Section 3.2 describes how those models are developed.

### 3.2. DNNs for Predicting Time-Varying Features

Both LSTM and CNN are implemented on the proposed fuzzy clustering-based DNN since they have been developed for power forecasting when time-varying features such as past weather, load consumption, climate and meteorological variables are given [17,28,29,30,31,33,34,36].

#### 3.2.1. Long Short-Term Memory Network

**Figure 4:**
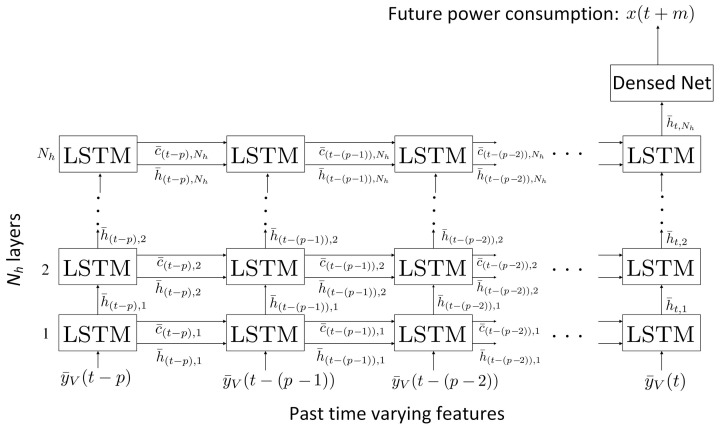
LSTM layer.

The LSTM network is suitable for time-series predictions since it benefits from long-term memory cells [56]. The LSTM network in Figure 4 is developed to forecast future load consumption, x(t+m), with *m* time units ahead, when the past time-varying features, ${\bar{y}}_{V}\left(t-p\right)$, ${\bar{y}}_{V}\left(t-\left(p-1\right)\right)$,… and ${\bar{y}}_{V}\left(t\right)$, are given. *p* denotes the number of temporal lags. The LSTM network consists of $N_{h}$ layers: an input layer which feeds in the past time-varying features in multi-dimensions, an LSTM layer with (p+1) neurons and a dense net which determines x(t+m) at the last layer. Each LSTM neuron is fed with (N−C) past time-varying features.

The LSTM nodes in Figure 4 are interconnected in order to update the neuron states with previous inputs. Each LSTM neuron has two inputs, namely previous short-term state, ${\bar{h}}_{t-(p-i),j}$, and previous long-term state, ${\bar{c}}_{t-(p-i),j}$, where 0≤i≤(p−1) and $1\leq j\leq N_{h}$. It also has two outputs, namely future short-term state, ${\bar{h}}_{t-(p-i)+1,j}$, and future long-term state, ${\bar{c}}_{t-(p-i)+1,j}$. The LSTM neurons select some of the previous short-term state and long-term state and pass those to the later LSTM neurons. At the last layer, the dense net forecasts x(t+m) by combining the values of all forecasting elements in ${\bar{h}}_{t,N_{h}}$.

**Figure 5:**
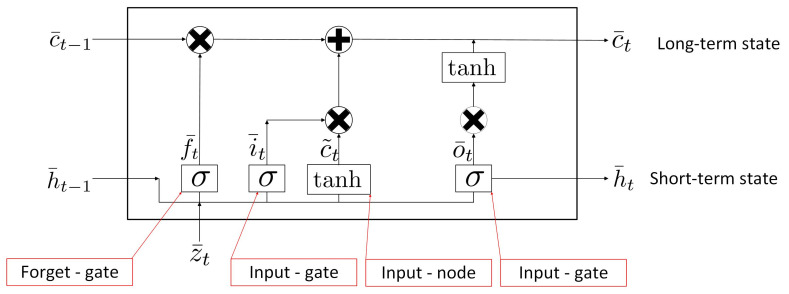
LSTM neuron.

Figure 5 illustrates the computations of how the LSTM neuron manipulates the previous and the future short- and long-term states. To simplify the state expression, the hidden layer index is omitted. The previous and future short-term states are denoted as ${\bar{h}}_{t-1}$ and ${\bar{h}}_{t}$, respectively; the previous and future long-term states are denoted as ${\bar{c}}_{t-1}$ and ${\bar{c}}_{t}$, respectively. The figure shows that the LSTM neuron consists of a main connected layer and three gate controller layers. The upper layer involves a control state which computes the future long-term state, ${\bar{c}}_{t}$, by analysing the current input gate, ${\bar{z}}_{t}$, previous short-term state, ${\bar{h}}_{t-1}$, and previous long-term state, ${\bar{c}}_{t-1}$. The lower layer involves ${\bar{f}}_{t}$ with the forget gate, ${\bar{i}}_{t}$ with the input gate, ${\tilde{c}}_{t}$, with the input node and ${\bar{o}}_{t}$ with the output gate. The LSTM states are changed by the three gate operations, such as by removing, writing or reading. The computations for ${\bar{f}}_{t}$, ${\bar{i}}_{t}$, ${\tilde{c}}_{t}$, ${\bar{o}}_{t}$, ${\bar{c}}_{t}$ and ${\bar{h}}_{t}$ are performed by (17a) to (17f), respectively:

(17a) $$\begin{array}{cccc} \; & \mathrm{Forget}\mathrm{gate}: & \; & {\bar{f}}_{t}=\sigma \left(W_{zf}^{T}\times {\bar{z}}_{t}+W_{hf}^{T}\times {\bar{h}}_{t-1}+{\bar{b}}_{f}\right) \end{array}$$

(17b) $$\begin{array}{cccc} \; & \mathrm{Input}\mathrm{gate}: & \; & {\bar{i}}_{t}=\sigma \left(W_{zi}^{T}\times {\bar{z}}_{t}+W_{hi}^{T}\times {\bar{h}}_{t-1}+{\bar{b}}_{i}\right) \end{array}$$

(17c) $$\begin{array}{cccc} \; & \mathrm{Input}\mathrm{node}: & \; & {\tilde{c}}_{t}=\tanh\left(W_{zc}^{T}\times {\bar{z}}_{t}+W_{hc}^{T}\times {\bar{h}}_{t-1}+{\bar{b}}_{c}\right) \end{array}$$

(17d) $$\begin{array}{cccc} \; & \mathrm{Output}\mathrm{gate}: & \; & {\bar{o}}_{t}=\sigma \left(W_{zo}^{T}\times {\bar{z}}_{t}+W_{ho}^{T}\times {\bar{h}}_{t-1}+{\bar{b}}_{o}\right) \end{array}$$

(17e) $$\begin{array}{cccc} \; & \mathrm{Long}-\mathrm{term}\mathrm{state}: & \; & {\bar{c}}_{t}={\bar{f}}_{t}⊗{\bar{c}}_{t-1}+{\bar{i}}_{t}⊗{\tilde{c}}_{t-1} \end{array}$$

(17f) $$\begin{array}{cccc} \; & \mathrm{Short}-\mathrm{term}\mathrm{state}: & \; & {\bar{h}}_{t}={\bar{o}}_{t}⊗\tanh{\bar{c}}_{t-1} \end{array}$$

where σ denotes the logistic activation function; $W_{zf}^{T}$, $W_{zi}^{T}$, $W_{zc}^{T}$ and $W_{zo}^{T}$ are the weight matrices of the four gates connecting to ${\bar{z}}_{t}$; $W_{hf}^{T}$, $W_{hi}^{T}$, $W_{hc}^{T}$ and $W_{ho}^{T}$ are the weight matrices of the four gates connecting to the previous short-term state $h_{t-1}$; $b_{f}$, $b_{i}$, $b_{c}$, and $b_{o}$ are the bias terms for the four gates.

The input gate and input node decide which parts of input, ${\bar{z}}_{t}$, are added to the long-term state, ${\bar{c}}_{t}$, after the forget gate, ${\bar{f}}_{t}$, stores the important part of ${\bar{z}}_{t}$ which needs to be kept. The output gate generates ${\bar{o}}_{t}$, which decides which parts of ${\bar{z}}_{t}$ need to be output for the current time. ${\bar{f}}_{t}$, ${\bar{i}}_{t}$ and ${\bar{o}}_{t}$ are the outputs of the σ function ranged from 0 to 1. $\tilde{c_{t}}$ is the output of the tanh function, which is between −1 and 1. After the input sequence is processed by the gate operations, the long-term memory, ${\bar{c}}_{t}$, and short-term memory, ${\bar{h}}_{t}$, are passed to the next or upper LSTM neurons.

#### 3.2.2. Convolution Neural Network

**Figure 6:**
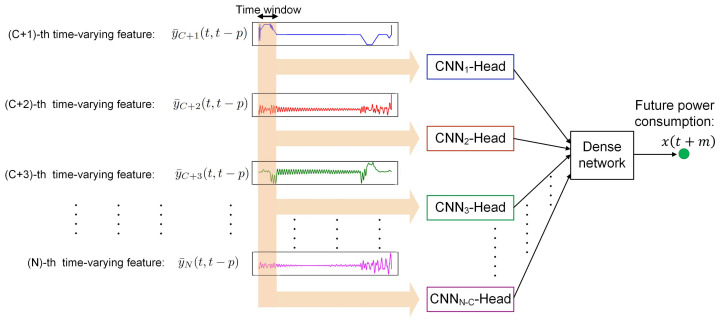
CNN framework [58].

Despite the LSTM, CNNs are suitable for predicting one-dimensional time-series data. Since sequential time-series data make up a one-dimensional image, a window-based convolution operation can be used to extract useful information [57]. Figure 6 illustrates the proposed CNN framework, which is a multi-head convolution network [58,59]. The framework consists of many CNN heads, which are developed for time-series prediction. The time series of each time-varying feature is processed by a CNN head. Since the time-varying features are indexed from (C+1) to *N*, the $i^{th}$ time-varying feature within a window between *t* and t−p, namely ${\bar{y}}_{I}\left(t,t-p\right)$ in (18), is processed by a${\mathrm{CNN}}_{\left(i-C\right)}-$ Head, where *t* is the current time and (t−p) is the past time with *p* sample lag and (C+1)≤i≤N.

(18) $${\bar{y}}_{i}\left(t,t-p\right)=\left\{y_{i}\left(t\right),y_{i}\left(t-1\right),\ldots ,y_{i}\left(t-p\right)\right\}$$

Each ${\mathrm{CNN}}_{\left(i-C\right)}$-Head is responsible for capturing useful information from ${\bar{y}}_{i}\left(t,t-p\right)$, which is correlated with the future load consumption, x(t+m). Since all ${\bar{y}}_{i}\left(t,t-p\right)$ have different natures and scales, each ${\bar{y}}_{i}\left(t,t-p\right)$ can be processed independently and useful information from each feature can be captured. The individual prediction of each ${\mathrm{CNN}}_{\left(i-C\right)}$-Head is gathered by a dense network in order to predict the future load consumption, x(t+m).

**Figure 7:**
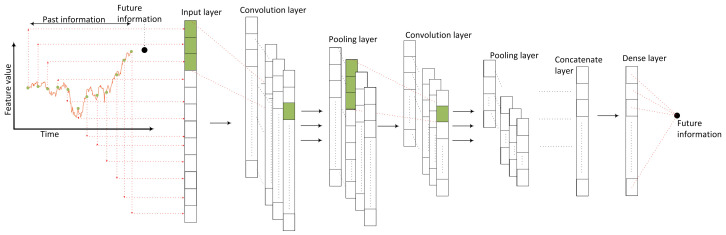
CNN-Head.

The CNN head in Figure 7 consists of an input layer, several convolution layers, several pooling layers, a concatenate layer and a dense layer. The input layer feeds in the time-varying feature, ${\bar{y}}_{i}\left(t,t-p\right)$. The convolution layer extracts important information from ${\bar{y}}_{i}\left(t,t-p\right)$. Each convolution layer consists of multiple sliding windows which scan input time series. The sliding window extracts useful information from the time series by capturing repeated patterns at different regions of the time series. Since the sliding windows in the convolution layer focus on the corresponding features, useful information from each feature can be kept. An activation function is applied to the convolution output to learn the nonlinear patterns of each feature. The pooling layer is used after the convolution layer to reduce the time-series size. After several convolutions and pooling operations, the processed time series is concatenated and is passed to the dense layer. The future information is passed to the dense network at the CNN framework in Figure 6 in order to predict the future load consumption, x(t+m).

## 4. Forecasting Performance Evaluations

This section presents the validation results obtained by the proposed fuzzy clustering-based DNNs, namely fuzzy LSTM and fuzzy CNN, which are integrated with the fuzzy clustering with the LSTM network in Section 3.2.1 and CNN in Section 3.2.2, respectively. Section 4.1 presents the load consumption data, which is used to evaluate the forecasting performance. Section 4.2 discusses how the fuzzy LSTM and fuzzy CNN are implemented. Section 4.3 presents the forecasting results.

### 4.1. Load Consumption Data

The performance of the proposed fuzzy LSTM and fuzzy CNN paper is evaluated by Miller’s dataset, which is used for developing load consumption predictors or for large building energy anomaly detection [47,48]. The data used for evaluating the proposed models was collected from two sites, City Building in Cardiff (City-Build) and University College London (University). The data were collected from 2016 to 2017. The data collected in 2016 are used to develop the models and those collected in 2017 are used to validate the prediction capabilities of the models. The numbers of buildings in City-Build and University are 89 and 51, respectively. Hourly meter reading data were captured from power meters installed in the two sites. Each building has one or more power meters measuring load consumption. The total hourly load consumption in a building is the sum of meter readings captured by all the installed meters. The buildings have various purposes, such as education, office and entertainment. The portions of building purposes are summarized in Table 1.

Each building has a corresponding weather data file which is recorded with hourly data for outdoor temperatures, humidity, cloud coverage and weather conditions. Those weather data influence load consumption. Those hourly weather data were collected from the National Center for Environmental Information (NCEI) National Oceanic and Atmospheric Administration (NOAA) Integrated Surface Database (ISD) (https://www.ncei.noaa.gov/products/land-based-station/integrated-surface-database). The dataset is used to develop the proposed fuzzy LSTM and fuzzy CNN, which consist of forecasting features collected from different domains, namely building, weather and calendar, and load consumption, as shown in Table 2.

In total, there are fourteen features. Two are in the building domain, seven are in the weather domain, four are in the calendar domain and one is in the load consumption domain. Both time-invariant features [39] and time-varying features [60] are correlated to load consumption.

Since some data are missing during data collection, data analysis cannot be performed by statistical or analytical tools [47]. Insertion and estimation of missing values are necessary prior to developing prediction models. Interpolations are performed to estimate missing values for the time-varying features. The missing values are inserted with the closest neighbour values. To improve the robustness of the model, data standardization is performed for each feature in the dataset. Data standardization in (19) is applied for each forecasting feature to ensure that data is internally consistent and also that the effect of outliers is reduced.

(19) $$\tilde{x}=\frac{\left(x-\bar{x}\right)}{\sigma }$$

where $\bar{x}$ and σ are the mean and standard derivation of the forecasting feature in the dataset, respectively.

### 4.2. Implementation of Forecasting Models

All algorithms are coded in python scripts and are implemented by a HP ZB 15G7 computer with 32 GB memory and a RTX 3000 GPU 6 GB card. The prediction models are all developed by the TensorFlow module. The prediction models are developed to forecast the future load consumption an hour ahead using a time window of 24 h from the current time to the past 23 h. Since an accurate amount of fossil fuel needs to be reserved hourly, this short-term prediction is necessary for fossil fuel power generators. Insufficient fossil fuel generates insufficient power to users.

The fuzzy c-mean clustering in Algorithm 1 is implemented to generate the centres of the fuzzy clusters, where the dataset of the time-invariant features are used. The fuzzy deep learning in Algorithm 2 is used to generate the prediction models, either the fuzzy LSTM or the fuzzy CNN, when the extra dataset of time-varying features is given. The threshold values of both fuzzy LSTM and fuzzy CNN are set at 0.5. After the fuzzy LSTM and fuzzy CNN models are developed by the training dataset collected in 2016, the test dataset collected in 2017 is used to validate the prediction capability of the developed models.

The proposed fuzzy LSTM in Figure 4 is implemented with 12 time-varying features. The LSTM network has an input layer, an LSTM hidden layer and a dense layer as the output layer. The input layer has 24 LSTM neurons, where each LSTM neuron is connected to a sample of the 24 h window. The second layer has 32 LSTM neurons and the last LSTM neuron generates the future prediction for the corresponding feature. A dense block is set at the output layer, where its inputs are the feature predictions and its output predicts the future load consumption.

The proposed fuzzy CNN in Figure 6 is implemented with the 12 individual CNN-Heads in Figure 7, where the input of each CNN-Head is the time-varying feature. In each CNN-Head, the first layer is the input layer, which is connected with the time series of a 24 time-sample window. The second layer is the convolution layer with 32 convolution filters, of which each filter has a window size of 3. The third layer is the pooling layer, with 32 max-pooling filters. The fourth layer consists of 32 concatenation filters, of which each concatenation filter concatenates the outputs of a max-pooling filter. The fifth layer has a single concatenation filter which concatenates the outputs of the 32 concatenation filters. The last dense layer processes the concatenated outputs and predicts the future information. Each individual CNN-Head generates the future information for each time-varying feature. The outputs from the 12 individual CNN-Heads are gathered by the dense network and the future load consumption is predicted.

### 4.3. Numerical Results for STLF

#### 4.3.1. Clustering of Time-Invariant Features

**Figure 8:**
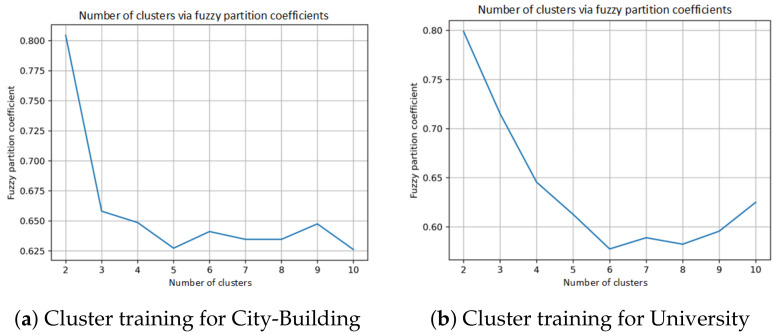
Cluster training.

The FCM in Algorithm 1 is used to determine the cluster centres of users with respect to the time-invariant features, namely Building size and Flow count, where the training dataset is used to determine the clusters. The FCM algorithm first determines the optimal number of centres, which achieves the largest fuzzy partition coefficient. The cluster centres with the largest fuzzy partition coefficient are selected and are implemented to select relevant users. The corresponding time-varying data in the same cluster are used to develop the STLF model. The numbers of clusters and the corresponding fuzzy partition coefficients are shown in Figure 8a,b for City-Building and University, respectively. Both figures show that the highest fuzzy partition coefficients can be achieved for both City-Building and University when the numbers of clusters are two. The user data in each cluster are used to develop a STLF model. Hence, two models are developed for these two clusters, respectively.

**Figure 9:**
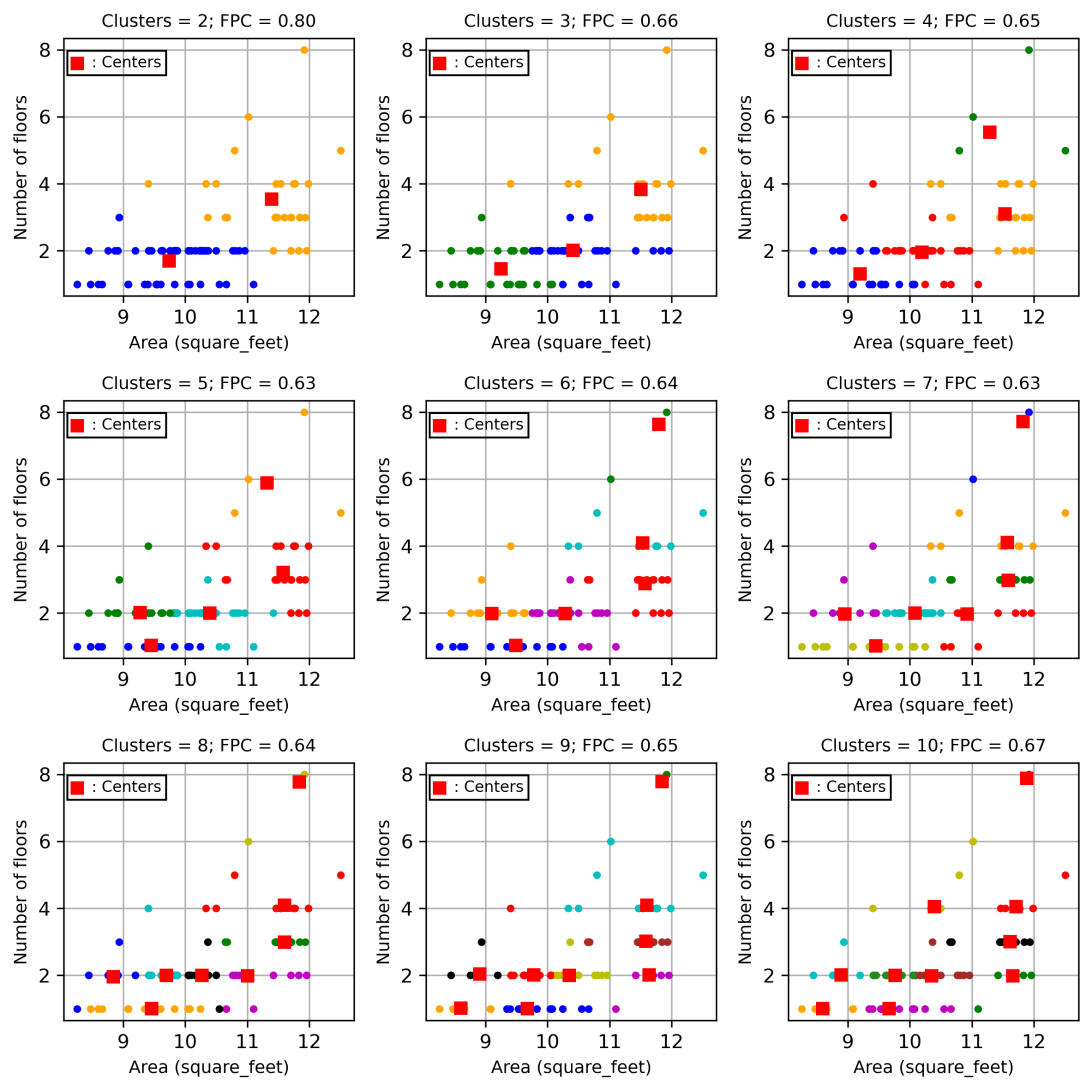
Clusters for City-Building.

**Figure 10:**
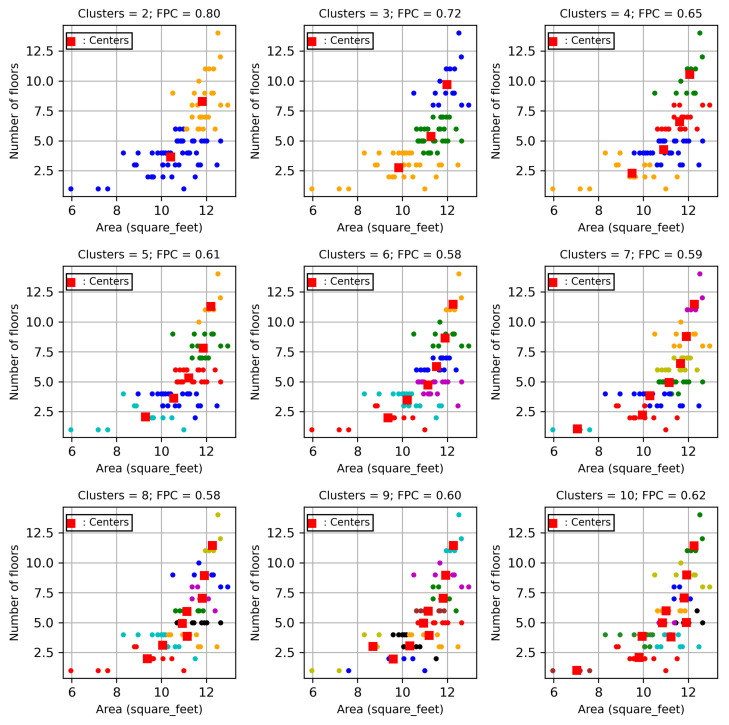
Clusters for University.

The clustering plots for City-Building and University are shown in Figure 9 and Figure 10, respectively. The figures show that the users for both City-Building and University are distributed on the relevant clusters with respect to numbers of floors and areas of the users. The figures show that the various cluster numbers from 2 to 9 are used to partition the users. For example, the top left subplot in Figure 9 shows that the users are distributed with two clusters. The two cluster centres are illustrated by two red squares. The two classes of users are illustrated with blue and yellow colours for the two clusters, respectively. Users which are closer to the lower centre are labelled with blue; users which are closer to the upper centre are labelled with yellow. Similarly, the top middle subplot shows how the users are distributed, with three clusters of each cluster having a centre with a square label. Three clusters contain users which are indexed with green, blue and yellow colours. Based on the cluster plot, we can identify which users belong to which clusters. When a new user moves to the site, the cluster plot can be used to identify which cluster contains the new user.

**Figure 11:**
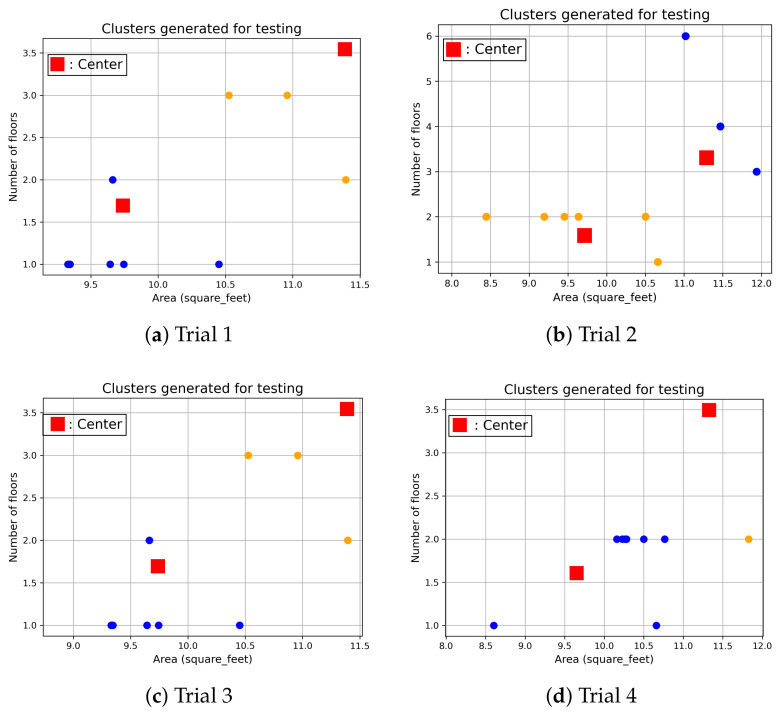
Cluster validations for City-Building.

**Figure 12:**
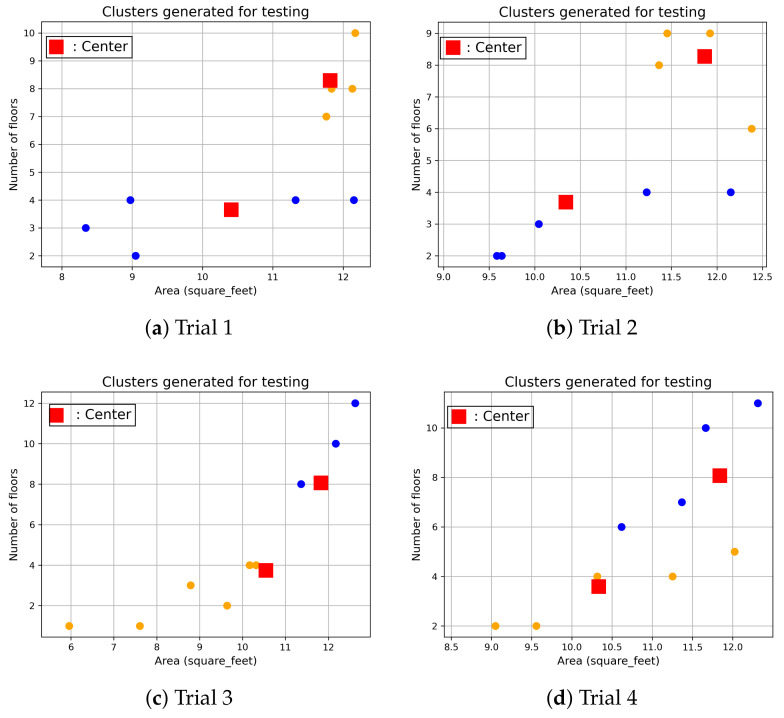
Cluster validations for University.

Figure 11 shows the clustering results of the test dataset for City-Building, and nine new users are randomly selected from the test dataset. The nine new users are classified by the clusters which are developed by the training dataset. The new users in the test dataset are not included to develop the clusters, and those new users are excluded in the training dataset. Four trials are conducted. The figures show that new users with similar numbers of floors and areas are classified on the cluster, where the cluster centre is close to the user features. Similar results can be found when clustering new users for University. Figure 12 shows that new users with similar numbers of floors and areas are closer to their corresponding centres. Training data of users in the same cluster are used to develop a single forecasting model. Since there are two clusters, two forecasting models are developed based on the user data from each cluster. When a new user is moved to University, the new user is classified into one of the clusters. The corresponding forecasting model is used to predict the future load consumption of this new user. Since existing users with similar behaviours are used to develop the model, more accurate prediction results can be achieved. Those forecasting results are presented in Section 4.3.2.

#### 4.3.2. Load Consumption Forecasting

**Figure 13:**
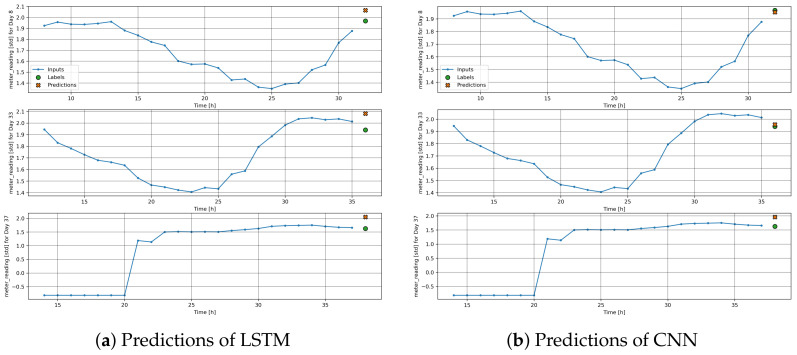
Predictions of LSTM and CNN for Day 8, Day 33 and Day 37 (standardized values).

**Figure 14:**
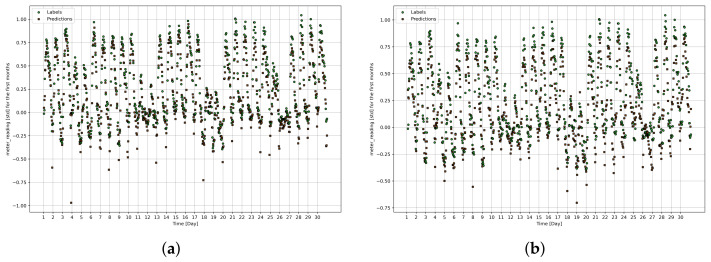
Actual load consumption (i.e., labels) and predictions of LSTM and CNN for the first month for building ID 684 (standardized values). (**a**) Actual load consumption (labels) and predictions of LSTM for building ID 684, (**b**) Actual load consumption (i.e., labels) and predictions of CNN for building ID 684.

Figure 13a,b show the prediction results obtained by the fuzzy LSTM and fuzzy CNN, respectively on Day 8, Day 33 and Day 37 of year 2017 for building ID 684, which is one of the buildings in City-Building. The results show the predictions with different time windows from 8 to 32, from 12 to 36 and from 14 to 38. All figures show predictions of an hour ahead. We can see that predictions are close to the actual load consumption when samples of the past 24 h are used for the predictions. Figure 14a,b show the predictions obtained by the fuzzy LSTM and fuzzy CNN for the first month for building ID 684, respectively. The predictions and the actual load consumption (i.e., labels) are represented by green and red points, respectively. The results show that the predictions are generally close to the actual load consumption. In addition, the figures show that load consumption is higher at the beginning of the day, compared to that at the end of the day. The results indicate that higher load consumption is generally required during the mornings and the afternoons.

Cross validation with 20 trials is performed to evaluate the performance of the prediction models. The performance of the proposed fuzzy LSTM and fuzzy CNN are compared with the commonly used LSTM and CNN, namely non-fuzzy LSTM and non-fuzzy CNN. Unlike the proposed methods, the non-fuzzy LSTM and non-fuzzy CNN are developed by modelling both time-varying and time-invariant features, and clustering is not performed on time-invariant features. These experiments validate whether or not the prediction accuracy can be enhanced by the clustering of time-invariant features and by solely modelling time-varying features. Nine building IDs are randomly selected from the test dataset, and those selected building IDs are excluded in the training dataset to develop the prediction models. Due to the page limitation, the results of all trials cannot all be illustrated. We present the first four trials.

**Figure 15:**
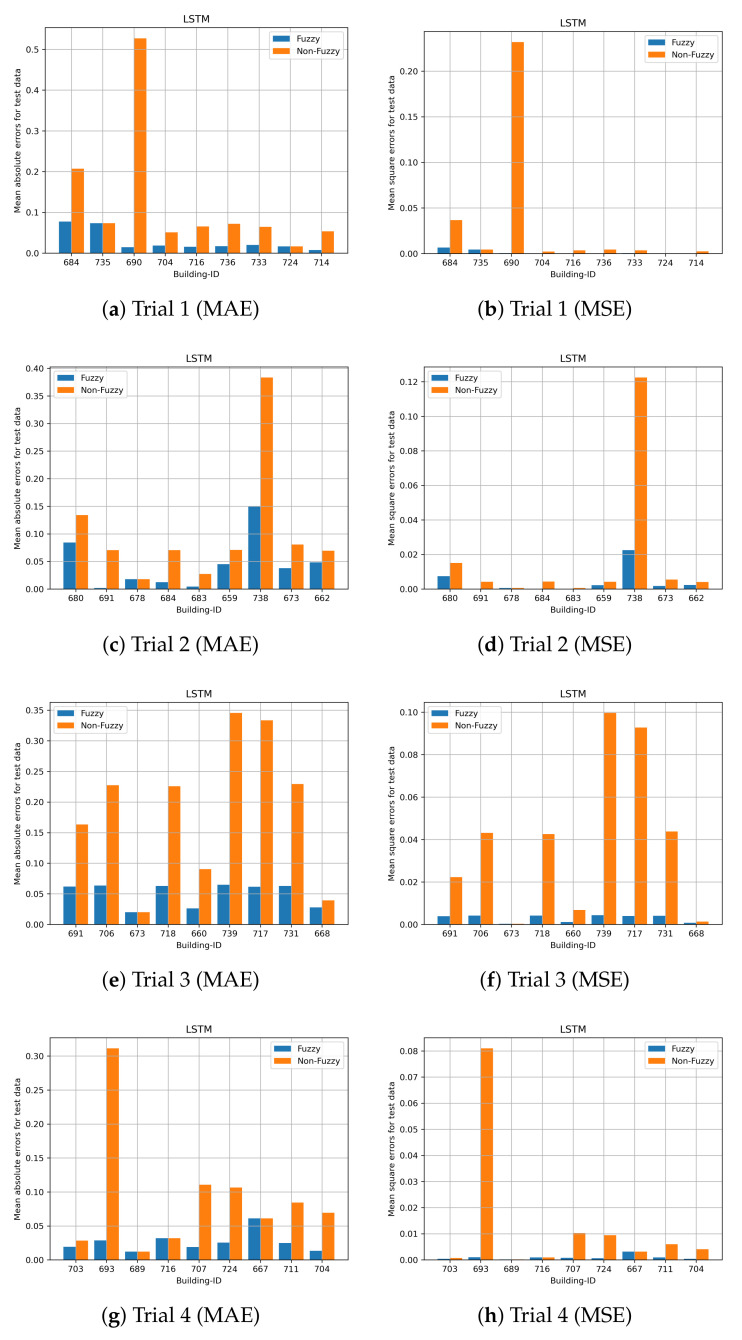
Site ID 1 with LSTM predictions (std error).

**Figure 16:**
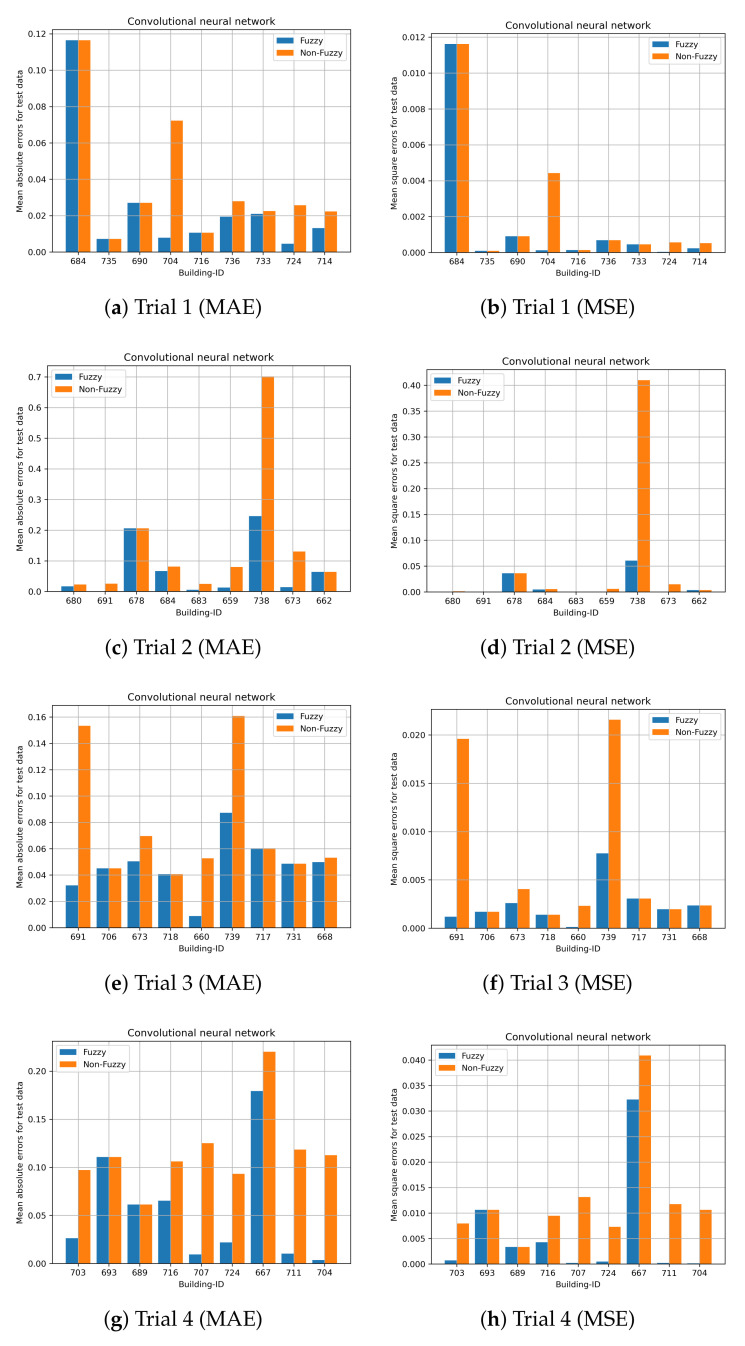
Site ID 1 with CNN predictions (std error).

**Figure 17:**
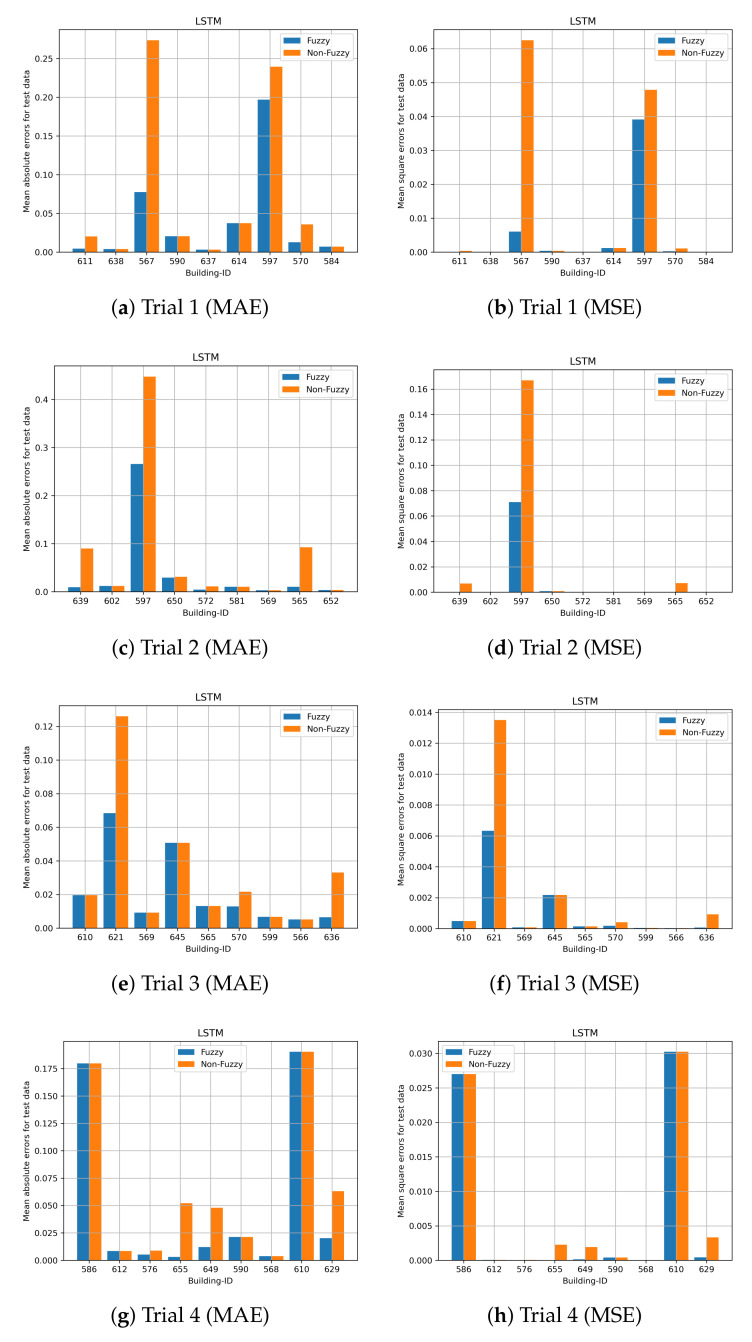
Site ID 4 with LSTM predictions (std error).

**Figure 18:**
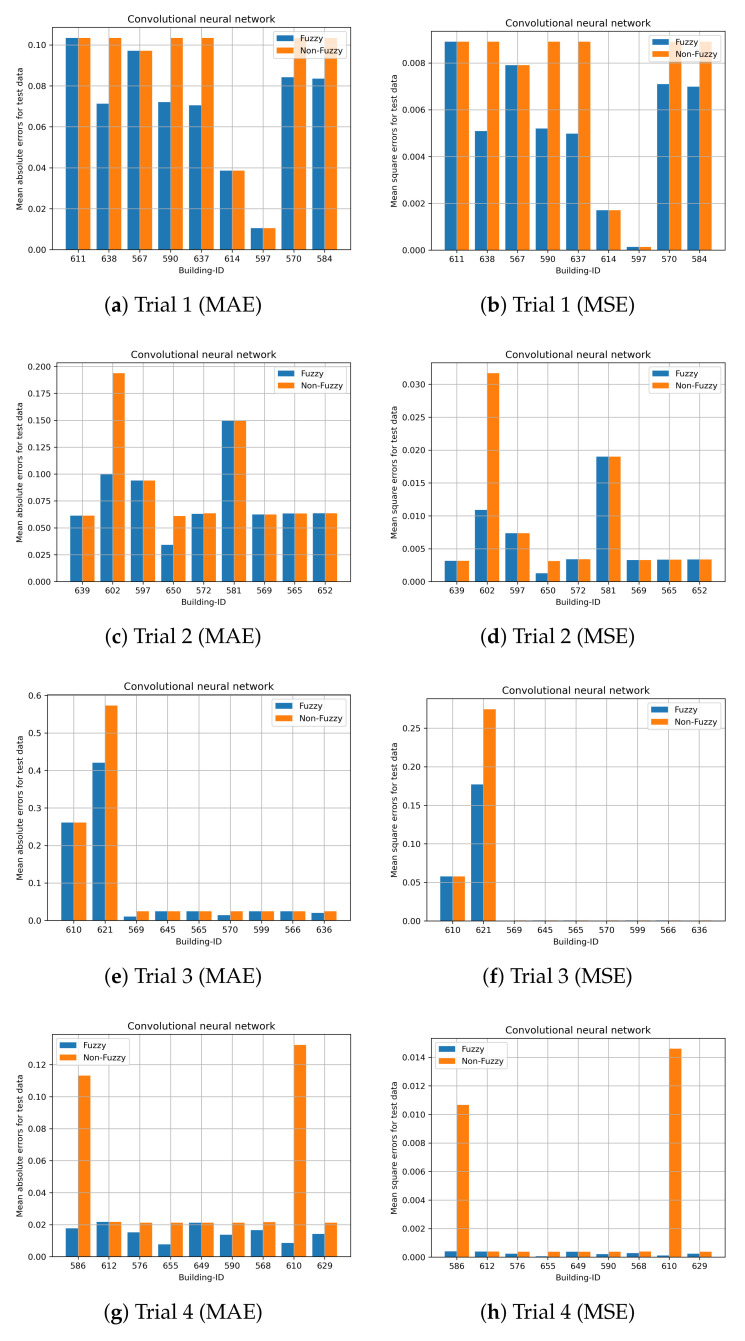
Site ID 4 with CNN predictions (std error).

The prediction results for the first four trials obtained by fuzzy LSTM and fuzzy CNN for City-Building are shown in Figure 15 and Figure 16, respectively. The nine building IDs which have been used for testing are shown in the x-axis. Those nine building IDs are used for validations and they are not included to develop the prediction models. Figure 16 shows both the mean absolute errors and mean square errors for the standardized load consumption. The results show that, generally, the proposed fuzzy CNN and fuzzy LSTM are able to obtain smaller errors compared with the non-fuzzy LSTM and non-fuzzy CNN. Both non-fuzzy approaches use both time-varying and time-invariant features to perform the predictions. Hence, more accurate results can generally be achieved by the proposed methods. Similar results can be found on University in Figure 17 and Figure 18 for the proposed fuzzy LSTM and fuzzy CNN, respectively. Generally, the proposed fuzzy LSTM and fuzzy CNN are able to obtain smaller prediction errors, although the same prediction errors are obtained by some building IDs. Table 3 shows the mean MAE and MSE obtained by the proposed fuzzy models and the non-fuzzy models. It shows further that the proposed fuzzy models are able to achieve smaller MAE and MSE; hence, more accurate predictions can be achieved. For the validation, a new user is first classified to a cluster, which has similar behaviours as the new user. The load consumption of the new user is predicted based on the model which is particularly developed to the classified cluster. Therefore, more accurate predictions can generally be achieved by the proposed fuzzy models.

## 5. Conclusions

In this paper, a novel STLF approach, namely the fuzzy clustering-based DNN, was proposed by integrating both time-varying and time-invariant features. The proposed fuzzy clustering-based DNN overcomes the limitation of commonly used STLF approaches which only consider time-varying features and ignore time-invariant features. The proposed approach uses the fuzzy c-means algorithm to group users with similar time-invariant features. DNN models do not need to learn the time-invariant features, and each DNN model only predicts time-varying dynamics in the same cluster which has similar time-invariant features. Both time-invariant and time-varying features are considered by the proposed method. Hence, more accurate predictions are likely to be achieved by the DNN models. The performance of the proposed method was evaluated by Miller’s data captured from City-Building and University with 140 buildings, which include both time-varying features and invariant time features. The experimental results obtained by the proposed method were compared with the commonly used DNN models, including LSTM and CNN. The results showed that the proposed method outperformed the LSTM and CNN; smaller mean square and mean absolution errors were achieved by the proposed method. Particularly smaller prediction errors were achieved for new users which have not been trained by the models. This paper only implemented the proposed fuzzy clustering-based DNN to perform STLF. Long-term load dynamics are also correlated to both time-varying features and time-invariant features. As well as using the proposed fuzzy clustering-based DNN for STLF, the proposed method can also be applied to long-term load forecasting in the electricity market. We will use the Miller’s data captured in 2016 to develop the models and will use the data captured in 2017 to validate the trained models. Long-term load forecasting can be performed in each season of 2017. Along with the Miller’s data, data recently captured from smart grid systems [61,62] will also be used to develop the models. The prediction capability of the proposed method can be further validated.

## Figures and Tables

**Table 1:** Portions of building purposes.

Purpose	Percentages
Education	37.71
Office	18.77
Entertainment/public assembly	12.47
Other	10.64
Lodging/residential	10.27
Public services	10.15

**Table 2:** Data features.

Feature Index	Forecasting Feature	Description of the Data (Range/Unit)	Domain	Time Nature
1	Building size	Floor area of building in square feet (564 to 1360 feet^2^)	Building	Time-invariant
2	Floor count	Minimum and maximum numbers of floors are 2 and 16, respectively	Building	Time-invariant
3	Air temperature	The temperature of the air from −10.6 to 47.2 °C	Weather	Time-varying
4	Cloud coverage	Portions of the sky covered in clouds from 0 to 9 oktas	Weather	Time-varying
5	Dewpoint temperature	A given parcel of air is cooled at a constant barometric pressure and water evaporation to saturate, −22.8 to 26.1 °C	Weather	Time-varying
6	Precipitation depth per an hour	The depth of liquid precipitation measured in an hour, −1 to 343 mm.	Weather	Time-varying
7	Sea level pressure	The air pressure relative to mean sea level, 973.5 to 1046.0 mb	Weather	Time-varying
8	Wind direction	The angle measured in a clockwise direction, between north and the direction of the blowing wind, 0 to 360°	Weather	Time-varying
9	Wind speed	The rate of horizontal travel of air past a fixed point, 0 to 18.5 m/s	Weather	Time-varying
10	Weekday	Sunday to Saturday indexed with 0 to 6	Calendar	Time-varying
11	Hour	24 h indexed with 0 to 23	Calendar	Time-varying
12	Month	January to December indexed with 1 to 12	Calendar	Time-varying
13	Timestamp	Year:Month:Date:Hour	Calendar	Time-varying
14	Previous 24 h load consumption	Load consumption ranges from 0 to 12 kW_*avg*_	Load consumption	Time-varying

**Table 3:** Test results for City Building and University (std error).

Sites	Methods	MAE	MSE
**City-Building**	Proposed fuzzy LSTM	0.051	0.003
	LSTM	0.218	0.042
	Proposed fuzzy CNN	0.151	0.026
	non-fuzzy CNN	0.415	0.174
**University**	Proposed fuzzy LSTM	0.075	0.005
	non-fuzzy LSTM	0.127	0.016
	Proposed fuzzy CNN	0.134	0.0176
	non-fuzzy CNN	0.187	0.0349

## Data Availability

Data are contained within the article.

## Nomenclature

**Abbreviation:**	
DNN	Deep neural network
LSTM	Long short-term memory network
CNN	Convolutionary neural network
FCM	Fuzzy C-means algorithm
STLF	Short-term load forecasting
**Load consumption forecasting:**	
*N*	Number of forecasting features
*C*	Number of time-invariant features
(N−C)	Number of time-varying features
x(t)	Load consumption at time *t*
e(t)	Noise resident at time *t*
x(t−p)	Past load consumption with *p* time sample lag
$\hat{x}\left(t+m\right)$	Predicted future load consumption with *m* time samples ahead
$y_{i}\left(t\right)$	$i^{th}$ forecasting feature
$\bar{y}\left(t\right)$	Forecasting feature vector containing all $y_{i}\left(t\right)$
$\bar{F}\left(t-p,t\right)$	Past information set containing $\bar{y}\left(t\right)$ and x(t) from time t−p to *t*
${\bar{y}}_{I}$	Forecasting feature vector containing time-invariant features
${\bar{y}}_{I}$	Time-invariant vector containing time-invariant features
${\bar{y}}_{V}\left(t\right)$	Time-varying vector containing time-varying features
${\bar{Y}}_{V}$	Time-varying set containing time-varying vectors

**User data:**	
*D*	Training dataset
*M*	Number of users sharing the grid systems
$d^{\left(i\right)}$	Data of the $i^{th}$ user
*n*	Number of samples in each $d^{\left(i\right)}$
$y_{j}^{\left(i\right)}\left(t\right)$	$j^{th}$ forecasting feature for the $i^{th}$ user at time *t*
$x^{\left(i\right)}\left(t-p\right)$	Past load consumption with *p* time sample lag for the $i^{th}$ user
${\bar{F}}^{\left(i\right)}\left(t-p,t\right)$	Past information set for the $i^{th}$ user with the window between (t−p) and *t*.
${\bar{y}}_{I}^{\left(i\right)}$	Time-invariant vector containing time-invariant features for the $i^{th}$ user
${\bar{y}}_{V}^{\left(i\right)}\left(t\right)$	Time-varying vector containing time-varying features for the $i^{th}$ user
${\bar{Y}}_{V}^{\left(i\right)}$	Time-varying set containing time-varying vectors for the $i^{th}$ user
**Clustering:**	
$N_{C}$	Number of clusters
${\hat{u}}_{k}\left({\bar{y}}_{I}^{\left(i\right)}\right)$	Membership of the $k^{th}$ cluster with respect to the time-invariant features of
	the $i^{th}$ users
${\hat{v}}_{k}$	Centre of the $k^{th}$ cluster
$\bar{V}$	Set of cluster centres
$V_{pc}$	Fuzzy partition coefficient
$d_{ik}$	the $\bar{A}$ Norm distance between ${\bar{y}}_{I}^{\left(i\right)}$ and the $k^{th}$ cluster centre
$\bar{A}$	Positive definite n×n weight matrix
$m_{f}$	Weighting exponent of the fuzzy clustering algorithm
${\hat{p}}_{j}$	Index vector indicating the time-invariant vectors in the $j^{th}$ cluster
$O_{j}$	Number of elements in the $j^{th}$ cluster
**Deep learning:**	
Θ	Deep neural network model
*W*	Weights of the DNN model
${\bar{h}}_{t}$	Short-term state of the long short-term memory network
${\bar{c}}_{t}$	Long-term states of the LSTM
${\bar{f}}_{t}$	Forget gate of the LSTM
${\bar{i}}_{t}$	Input gate of the LSTM
${\tilde{c}}_{t}$	Input node of the LSTM
${\bar{o}}_{t}$	Output gate of the LSTM
${\mathrm{CNN}}_{i}$-Head	CNN head for the ${\left(C+1\right)}^{th}$ time-varying feature in the CNN framework
